# Supplementary material for: Biosynthesized ZnO-NPs from Morus indica Attenuates Methylglyoxal-Induced Protein Glycation and RBC Damage: In-Vitro, In-Vivo and Molecular Docking Study
Source: Biomolecules. 2019 Dec 16;9(12):882. doi: 10.3390/biom9120882 (PMC6995520; doi:10.3390/biom9120882)
Supplement: Supplementary file 1 [file biomolecules-09-00882-s001.pdf]

biomolecules

MDPI

Supplementary Materials

Biosynthesized ZnO-NPs from *Morus indica* Attenuates Methylglyoxal-Induced Protein Glycation and RBC Damage: In-Vitro, In-Vivo and Molecular Docking Study

Satish Anandan [1], Murali Mahadevamurthy [2], Mohammad Azam Ansari [3], Mohammad A. Alzohairy [4], Mohammad N. Alomary [5], Syeda Farha Siraj [1], Sarjan Haluguddе Nagaraja [6], Mahendra Chikkamadaiah [2], Lakshmeesha Thimappa Ramachandrappa [7], Hemanth Kumar Naguvanahalli Krishnappa [2], Ana E. Ledesma [8], Amruthesh Kestur Nagaraj [2] and Asna Urooj [1,*]

1. Department of Studies in Food Science and Nutrition, University of Mysore, Manasagangotri, Mysuru, Karnataka 570006, India
2. Applied Plant Pathology Laboratory, Department of Studies in Botany, University of Mysore, Manasagangotri, Mysuru, Karnataka 570006, India
3. Department of Epidemic Disease Research, Institute for Research & Medical Consultations (IRMC), Imam Abdulrahman Bin Faisal University, P.O. Box 1982, Dammam 31441, Saudi Arabia
4. Department of Medical Laboratories, College of Applied Medical Sciences, Qassim University, Qassim 51431, Saudi Arabia
5. National Center for Biotechnology, Life Science and Environmental Research Institute, King Abdulaziz City for Science and Technology, P.O. Box 6086, Riyadh, Saudi Arabia
6. Department of Studies in Zoology, University of Mysore, Manasagangotri, Mysuru, Karnataka 570006, India
7. Department of Studies in Biotechnology, University of Mysore, Manasagangotri, Mysuru, Karnataka 570006, India
8. Centro De Investigación en Biofísica Aplicada y Alimentos, Universidad Nacional de Santiago del Estero (CIBAAL-UNSE-CONICET), FCEyT, RN 9, km 1125, CP 4206 Santiago del Estero, Argentina

* Correspondence: asnaurooj@foodsci.uni-mysore.ac.in

Table S1. Hemoglobin-δ-Gluconolactone (δ-Glu) assay.

Treatments		Inhibition of HbA1c (%)
Hb + δ-Gluconolactone + AG		45.89 ± 1.14 [a]
Hb + δ-Gluconolactone + ZnO-NPs	1 mg	1.81 ± 0.87 [d]
	2.5 mg	13.97 ± 1.30 [c]
	5 mg	26.54 ± 1.21 [b]
Hb+ δ-Gluconolactone + Zinc Nanopowder	1 mg	0.27 ± 0.48 [d]
	2.5 mg	0.53 ± 0.86 [d]
	5 mg	1.13 ± 0.97 [d]

Values are means of three independent replicates (n = 3) and ± indicate standard errors. Means followed by the same letter(s) within the same column are not significantly (p < 0.05) different according to Tukey's HSD.

Table S2. Inhibitory effect of biosynthesized ZnO-NPs from *M. indica* on protein glycation.

Treatments		Relative Intensity (a.u.)	
		**Fluorescence of Total AGEs**	**Fluorescence of Argpyrimidine**
BSA		508.66 ± 8.15[e]	312.15 ± 4.13[g]
BSA + MGO		1945.70 ± 38.08[a]	981.50 ± 7.07[a]
BSA + MGO + AG		569.90 ± 7.24[e]	365.23 ± 7.22[f]
BSA + MGO + ZnO-NPs	1 mg	1616.90 ± 13.89[c]	809.91 ± 7.58[c]
	2.5 mg	1369.70 ± 14.55[d]	705.08 ± 10.16[d]
	5 mg	1152.30 ± 37.42[d]	589.60 ± 9.95[e]
BSA + MGO + Zinc Nanopowder	1 mg	1838.00 ± 35.89[b]	976.87 ± 10.06[a]
	2.5 mg	1806.60 ± 52.02[b]	939.78 ± 9.22[b]
	5 mg	1795.10 ± 62.02[b]	934.09 ± 11.57[b]

Values are means of three independent replicates (n = 3) and ± indicate standard errors. Means followed by the same letter(s) within the same column are not significantly (p < 0.05) different according to Tukey's HSD.

Table S3. N-Acetylglycyl-lysine methyl ester (G.K.) peptide mediated ribose glycation with and without treatment.

**Treatments**	**Relative Intensity (a.u.)**
GKP + Ribose	743.19 ± 8.91[a]
GKP + Ribose + AG	290.41 ± 7.42[d]
GKP + Ribose + ZnO-NPs	398.41 ± 7.34[c]
GKP + Ribose + Zinc Nanopowder	693.16 ± 8.94[b]

Values are means of three independent replicates (n = 3) and ± indicate standard errors. Means followed by the same letter(s) within the same column are not significantly (p < 0.05) different according to Tukey's HSD.
